# Serum concentrations of IL-16 and its genetic polymorphism rs4778889 affect the susceptibility and severity of endometriosis in Nigerian women

**DOI:** 10.1186/s12905-023-02362-8

**Published:** 2023-05-11

**Authors:** Ochuwa Adiketu Babah, Oyesola Oyewole Ojewunmi, Chika Kingsley Onwuamah, Ifeoma Christiana Udenze, Akinniyi Adediran Osuntoki, Bosede Bukola Afolabi

**Affiliations:** 1 Department of Obstetrics & Gynaecology, Faculty of Clinical Sciences, College of Medicine, University , Hospital, PMB 12005, Surulere, Idi-Araba, Lagos, Nigeria; 2 DNA Laboratory, Sickle Cell Foundation Nigeria, Idi-Araba, Lagos, Nigeria; 3 School of Cancer and Pharmaceutical Sciences, King’s College, London, UK; 4 Centre for Human Virology and Genomics, Nigerian Institute of Medical Research, Lagos, Nigeria; 5 Department of Chemical Pathology, Faculty of Clinical Sciences, College of Medicine, University of Lagos/ Lagos University Teaching Hospital, Idi-Araba, Lagos, Nigeria; 6 Molecular Biology Unit, Department of Biochemistry, Faculty of Basic Medical Sciences, College of Medicine, University of Lagos, Lagos, Nigeria

**Keywords:** Endometriosis, Interleukin-6, Interleukin-16, Genes, Ascites, Genetic polymorphism, Pain, Nigeria

## Abstract

**Background:**

Endometriosis is the presence of active ectopic endometrial glands and stroma at other sites outside the uterine cavity. It is a common cause of chronic pelvic pain which is sometimes debilitating, and inflammation is one of the known triggers of endometriosis. Interleukins 6 and 16 (IL-6 and IL-16) are proinflammatory cytokines which play essential roles in inflammatory diseases. We therefore investigated the relationship between genetic polymorphisms of interleukins 6 and 16, and the development of endometriosis in Nigerian women.

**Method:**

One hundred and thirty (130) consenting women were consecutively enrolled, sixty-five (65) of whom had endometriosis and 65 age-matched women as reference group, surgically confirmed as not having endometriosis. Spectrophotometric determination of serum concentrations of Interleukins 6 and 16 was carried out and the genotyping of IL-6 (rs1800795) and IL-16 (rs4778889, rs11556218, rs4072111) genes were performed using TaqMan assays.

**Results:**

Serum IL-16 concentration was significantly higher in women with severe chronic pelvic pain compared to those with mild pain (*p* = 0.023). The C allele of rs4778889 was associated with endometriosis (OR: 1.80, 95% CI: 1.08 – 3.02, *p* = 0.024).

**Conclusion:**

Serum IL-16 and IL-16 rs4778889 may be important markers for endometriosis in Nigerian, and by extension, African women. Multicentre African studies would clarify this.

## What is already known?

*• *Interleukins play a vital role in the pain pathway.

*• *A change in serum interleukin concentration has been found in some studies to be associated with some clinical conditions whose manifestations include pain such as osteoarthritis, myocardial infarction, and Crohn's disease.

*• *Pain is a common manifestation of endometriosis.

*• *Endometriosis has been associated with polymorphisms in interleukin genes but with conflicting reports.

## What are the new findings from this study?

*• *The minor allele ‘C’ of IL-16 (rs4778889) is associated with endometriosis in Nigerian women with endometriosis.

*• *There is an association between serum concentration of IL-16 and pain severity in women with endometriosis.

## Introduction

Endometriosis is characterised by the presence of active ectopic endometrial glands and stroma at other sites outside the uterine cavity. It is a common condition and up to 71% of affected women usually report pain-related symptoms such as chronic pelvic pain and dysmenorrhoea [1]. It may predispose to infertility in 30 – 50% of women [2]. Diagnosis often presents a challenge, and many cases are diagnosed late [3]. Despite the numerous studies conducted on various aspects, the features of endometriosis remain challenging to manage [4, 5].

The role of interleukins as proinflammatory markers, autoimmune and immune modulators have led to exploration of their effect in the development of endometriosis. Several proinflammatory cytokines, namely interleukins 1, 6, 10 and 16, tumour necrosis factor-α and prostaglandin E have been postulated to play a role in the development of endometriosis [6–8].

IL-6 is a multifunctional cytokine with both pro- and anti-inflammatory properties. It is produced by different types of cells, including activated macrophages, monocytes, fibroblasts, activated T lymphocytes and B lymphocytes, and endothelial cells. IL-6 is involved in the control of homeostasis of cell processes, including lipid metabolism, mitochondrial activities, neuroendocrine system function and neuropsychological behaviour [9]. Comparatively, IL-6 presents low levels under normal conditions but is elevated when an event stimulates immune response. Some studies have suggested that IL-6 is a good marker for disease progression in endometriosis [10, 11].

The IL-6 gene is located on chromosome 7p21-24 and is composed of six exons and five introns. rs1800795 (-174*G* > *C*) is a common functional SNP in the promoter gene of IL-6, known to affect transcriptional activity and IL-6 concentrations [12, 13].

Interleukin-16 (IL-16), also known as a lymphocyte chemoattractant factor, is a multifunctional proinflammatory cytokine that performs an essential role in many immune and inflammatory responses. IL-16, through the peripheral blood mononuclear cells, stimulates the production of proinflammatory cytokines such as IL-6, IL-1β and TNF-α, which have been demonstrated to play a critical role in the pathogenesis of endometriosis [14]. Koga et al. have shown increased concentrations of IL-16 in the peritoneal fluid of patients with endometriosis and revealed that it may play a role in initiating or sustaining inflammatory responses in the peritoneal cavity.

The gene-encoding IL-16 is mapped to chromosome 15q26.3 in the human genome consisting of seven exons and six introns. IL-16 exists as a 631-amino acid precursor protein, Pro-IL-16, which is cleaved by caspase-3 to release the functionally active C-terminal domain, comprising 121 amino acids [15, 16]. Three common single nucleotide polymorphisms in IL-16 (rs4778889 T/C, rs11556218 T/G, and rs4072111) have been found to be associated with inflammatory diseases like asthma, Crohn's disease, cancer, and ischaemic stroke [17–20]. In a study conducted among Chinese women, the genotype and allele frequencies of rs4778889 T/C polymorphism were associated with endometriosis and pain phenotype [21].

Many studies have been conducted on interleukin 6 and 16 gene polymorphisms in women with endometriosis but considering the rarity of genetic research into benign gynaecological conditions in Africa, this study which to the best of our knowledge will be the first to be done in Africa, became a necessity.

This study aims to determine serum concentrations of IL-6 and IL-16 in women with endometriosis and investigate whether the genetic polymorphisms of Interleukin 6 (rs1800795G/C) and 16 (rs4072111C/T, rs11556218T/G, rs4778889T/C) are associated with endometriosis and endometriosis-related symptoms in Nigerian women.

## Patients and methods

This was a case–control study conducted between October 2019 and June 2021 at the Department of Obstetrics and Gynaecology of the College of Medicine of University of Lagos/ Lagos University Teaching Hospital (LUTH), Idi-Araba, Lagos, Nigeria.

All consenting women diagnosed as having endometriosis by laparoscopy, laparotomy, histology or who had shown clinical evidence of response to treatment for endometriosis were recruited as cases. Age-matched women who have had laparoscopy or laparotomy for other benign gynaecological conditions with no evidence of endometriosis at surgery, were recruited as reference group. Women in whom diagnosis was in doubt, or had adenomyosis, cancers, chronic medical illness such as renal disease, cardiovascular disease, diabetes mellitus or chronic infections were excluded from the study.

Participant's information was collected by direct questioning or from case notes using the proforma designed for this study. The information obtained included number of births, symptomatology, and family history of endometriosis or other genetic disorders. A numerical rating scale was used for pain assessment in those who reported chronic pelvic pain as a symptom. Pain score was classified as 0–5 mild, 6–7 moderate, and 8–10 severe [22]. For this study, we defined ascites as fluid accumulation within the peritoneal cavity clinically demonstrable by shifting dullness on percussion of the abdomen or peritoneal fluid accumulations of 500mls or more drained during abdominal paracentesis or other surgical procedures [23, 24]. Four millilitres of peripheral blood was collected from each participant by venepuncture. The collected blood samples were stored at -80 ^0^C until analysis. Spectrophotometric determination of serum Interleukins 6 and 16 concentrations was done using Ray Biotech ELISA kits (RayBiotech Life, Inc., GA, USA).

Genomic DNA was isolated from peripheral blood samples using a spin column DNA extraction kit (Jena Bioscience, Germany) according to the manufacturer's instructions**.** The DNA concentration was determined using a Nanodrop spectrophotometer One (Thermo Scientific, USA). At the genotyping facility, DNA concentration was determined using Qubit dsDNA reagent and Qubit 4 spectrophotometer. Where necessary, 10 ng/µL of each sample was prepared by dilution as genotyping performs better with similar amounts of sample DNA.

Genotyping of four single nucleotide polymorphisms was performed using TaqMan assays on a QuantStudio 5 real-time PCR system (Thermo Fisher Scientific, Singapore). Primers and master mixes for the TaqMan® SNP genotyping of IL-16 gene (rs4072111 C/T, rs11556218 T/G, and rs4778889 C/T) and IL-6 gene (rs1800795C/G) were obtained from Thermo Fisher Scientific (Table 1).

**Table 1:** SNP numbers and ordering codes

	ID	SNP_ID
1	rs1800795G/C	C___1839697_20 Catalog number: 4351379 SNP ID: rs1800795
2	rs4072111C/T	C____118300_20 Catalog number: 4351379 SNP ID: rs4072111
3	rs11556218T/G	C__25646461_40 Catalog number: 4351379 SNP ID: rs11556218
4	rs4778889T/C	C__31837550_10 Catalog number: 4351379 SNP ID: rs4778889

Briefly, the TaqMan genotyping assays come as 40X, and a working 20X is prepared, and analysis is performed according to the manufacturer’s instructions. The TaqPath ProAmp master mixes, with ROX to equilibrate background, were used to perform the replicate real-time PCR analysis. Twenty-five microlitre (25 µL) reactions were set-up on the standard 96-well QuantStudio 5, containing TaqPath ProAmp master mix (12.5 µL), working 20X TaqMan assay (1.25 µL), sample DNA (10 ng) and PCR-grade water. Each run had two–three no-template controls. The genotyping PCR template was used, with two holds for pre-read (60ºC for 30 s) and denaturation/enzyme activation (95ºC for 5 min), 40 cycles of denature (95ºC for 15 s), anneal/extend (60ºC for 60 s), and finally a post-read hold (60ºC for 30 s). Data collection was done at pre-read, end of each cycle and at post-read. Genotypes were analysed and read off the allelic discrimination plot.

### Sample size

A sample size of 128 participants (64 per group) was estimated to give this study a statistical power of 80% at a significance level of 5%, considering 20% attrition for missing data and other contingencies, using sample size calculator for genetic association case–control study accessed via http://osse.bii.a-star.edu.sg/calculation1.php as described in Mondry et al., 2006 [25].

### Statistical analysis

Results are presented as frequencies, percentages, and mean ± standard deviation. Test of normality was performed using Shapiro–Wilk test. Serum IL-6 and IL-16 concentrations deviated from the normal distribution and are presented as median [interquartile range]. Differences between continuous variables summarised as means were compared using an independent student's t-test, while serum IL-6 and IL-16 concentrations were compared using the Mann–Whitney U test or Kruskal–Wallis test where appropriate. Frequencies of the patients with endometriosis and the reference group were compared by chi-square test (χ^2^) or linear-by-linear association. Odds ratios (OR) and 95% confidence intervals (95%CI) were also calculated. All statistical analyses were conducted using SPSS version 27 for Windows and R software with *p*-value < 0.05 considered significant.

## Results

The mean age and body mass index of healthy women (32.8 ± 6.67 years old, 25.3 ± 4.38 kg/m^2^) compared to those with endometriosis (33.06 ± 6.78 years old, 24.4 ± 4.19 kg/m^2^) were not significant. Women with endometriosis had a significantly higher frequency of chronic pelvic pain, painful menstruation, painful intercourse, painful defaecation, and painful urination (*p* < 0.0001), compared to healthy women. Only one woman (1.5%) in the reference group had ascites compared to 22 (33.8%) in the case group. Pleural effusion (16.9%) was only observed in the case group. Thirty-five (53.8%) women with endometriosis had pelvic endometriosis while 29 (44.6%) and 1 (1.5%) had extra-pelvic and both pelvic/extra-pelvic endometriosis, respectively. Majority (93.8%) of women with endometriosis had no family history of endometriosis while 3 (4.6%) reported family history of endometriosis. Endometriosis was diagnosed by laparotomy (36.9%), clinical features (29.2%), and laparoscopy (27.7%); others were diagnosed by histology (3.1%) and thoracotomy (3.1%) (Table 2).

**Table 2:** Demographics and characteristics of study participants

Characteristics	Questionnaire answer	Reference group (*n* = 65)	Case (*n* = 65)	*p*-value
Age		32.8 ± 6.67	33.06 ± 6.78	0.817
BMI		25.3 ± 4.38	24.4 ± 4.19	0.842
Number of births		0–4	0–3	-
Chronic pelvic pain	NO	56 (86.2%)	20 (30.8%)	**< 0.0001**
	YES	9 (13.8%)	45 (69.2%)	
Painful menstruation	NO	34 (52.3%)	3 (4.6%)	**< 0.0001**
	YES	31 (47.7%)	62 (95.4%)	
Painful intercourse	NO	60 (92.3%)	32 (49.2%)	**< 0.0001**
	YES	5 (7.7%)	29 (44.6%)	
	I don’t know	-	4 (6.2%)	
Bleeding/site of bleeding	None	-	50 (76.9%)	-
	Umbilicus	-	15 (23.1%)	
Blood in stool	NO	64 (98.5%)	62 (95.4%)	0.703
	YES	-	3 (4.6%)	
	I don’t know	1 (1.5%)	-	
Painful defaecation	NO	65 (100%)	48 (73.8%)	**< 0.0001**
	YES	-	17 (26.2%)	
Blood in urine	NO	64 (98.5%)	62 (95.4%)	0.400
	YES	-	1 (1.5%)	
	I don’t know	1 (1.5%)	2 (3.1%)	
Painful urination	NO	64 (98.5%)	49 (75.4%)	**< 0.0001**
	YES	1 (1.5%)	15 (23.1%)	
	I don’t know	-	1 (1.5%)	
Convulsion during menstruation	NO	65 (100%)	64 (98.5%)	0.317
	I don’t know	-	1 (1.5%)	
Coughing up blood	NO	65 (100%)	63 (96.9%)	0.496
	YES	-	2 (3.1%)	
Ascites	NO	64 (98.5%)	43 (66.2%)	**< 0.0001**
	YES	1 (1.5%)	22 (33.8%)	
Pleural effusion	NO	65 (100%)	54 (83.1%)	**0.001**
	YES	-	11 (16.9%)	
Type of endometriosis	Pelvic	-	35 (53.8%)	-
	Extra-pelvic	-	29 (44.6%)	
	Both	-	1 (1.5%)	
No Symptom	NO	43 (66.2%)	64 (98.5%)	**< 0.0001**
	YES	22 (33.8%)	1 (1.5%)	
Family history of endometriosis	NO	65 (100%)	61 (93.8%)	0.056
	YES	-	3 (4.6%)	
	I don’t know	-	1 (1.5%)	
Method of diagnosis	Laparotomy	53 (94.6%)	24 (36.9%)	0.120
	Laparoscopy	3 (5.4%)	18 (27.7%)	
	Clinical features	-	19 (29.2%)	
	Histology	-	2 (3.1%)	
	Thoracotomy	-	2 (3.1%)	

Age and BMI are presented as Mean (± S.D.), number of births is presented as median (range), and others as frequency (percentage)

**Fig. 1:**
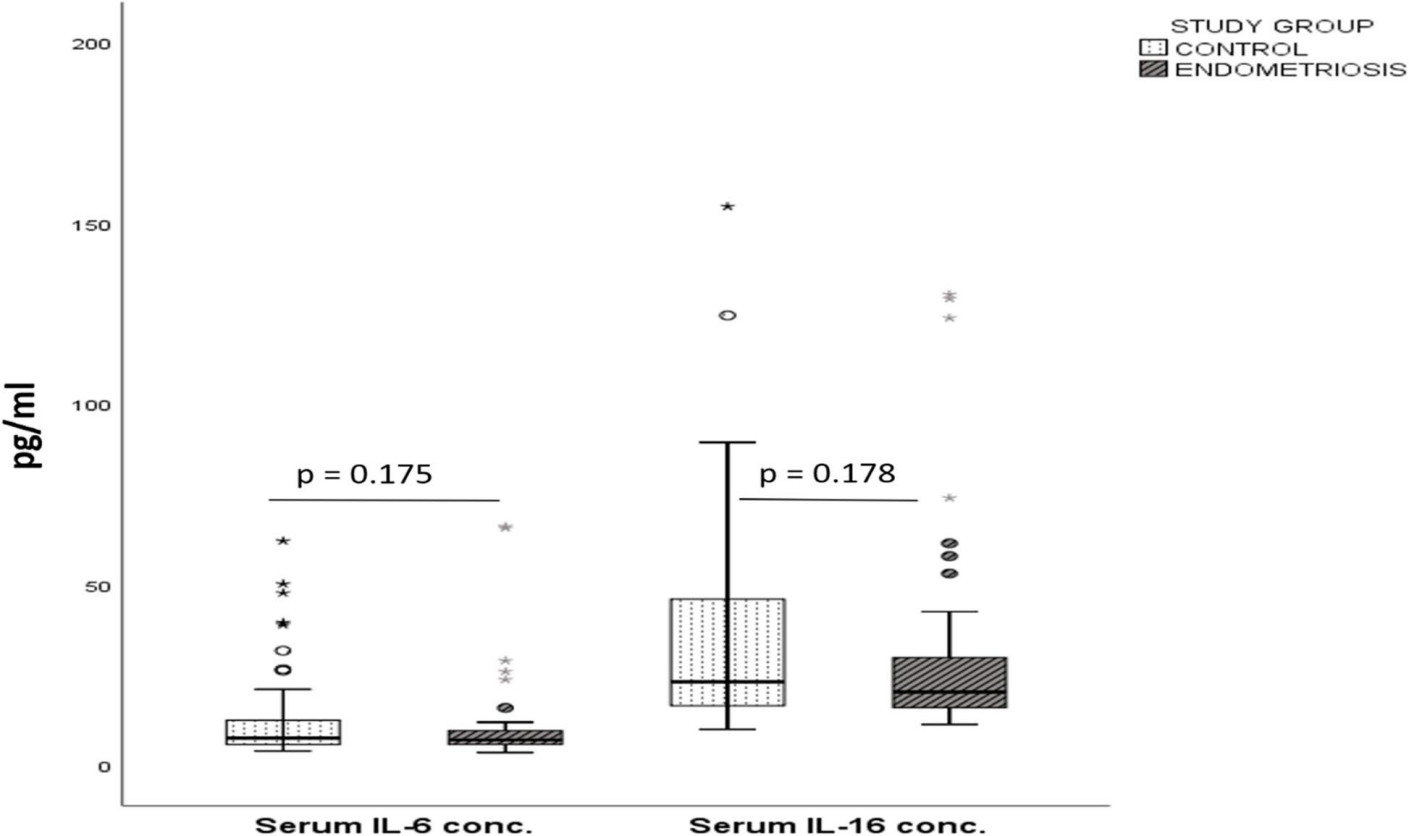
Serum concentrations of IL-6 and IL-16 concentration in the reference and case groups

There was no statistically significant difference in IL-6 or IL-16 serum concentration between the two groups. The median serum IL-6 for the reference group, 7.61[5.81 – 12.78] pg/ml was higher than the women with endometriosis, 7.07[5.84 – 9.67] pg/ml with *p*-value of 0.175. A similar pattern was observed with the median serum IL-16 for the reference group, 23.17[16.39 – 45.99] pg/ml and case, 20.33[15.99 – 29.90] pg/ml groups with *p*-value of 0.178 (Fig. 1).

There was no statistical significance in the IL-6 serum concentration distribution according to the pain score of women with endometriosis. The median IL-6 serum concentrations was found to be 6.5 [6.4 – 8.6]pg/ml in women with endometriosis who reported mild pain, 7.1 [6.1 – 11.3]pg/ml in those who reported moderate pain, and 7.1 [5.7 – 9.3]pg/ml in those who had severe pain.

**Fig. 2:**
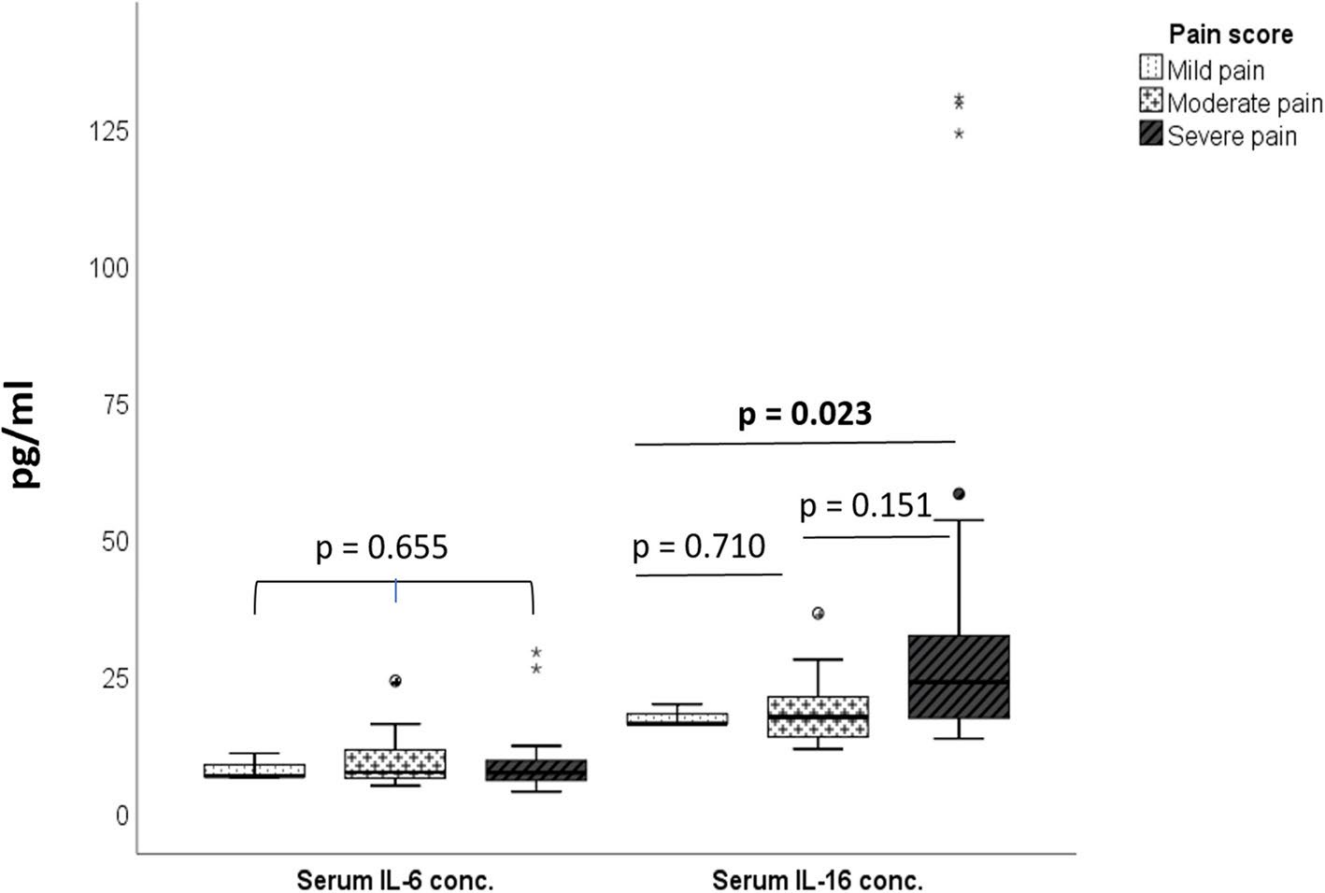
Serum concentrations of IL-6 and IL-16 according to pain score

IL-16 serum distribution in women with endometriosis was significantly higher (*p* = 0.023) in those with severe pain (23.5[17.1 – 32.0])pg/ml compared to those who had mild pain (16.0 [16.0 – 17.8])pg/ml. IL-16 serum concentrations in women with moderate pain (17.2 [13.6 -20.9])pg/ml were not significantly different from those with mild and severe pain (Fig. 2).

IL-6 (rs1800795) was monomorphic for both the reference and the case groups. All the polymorphisms investigated in the IL-16 were in hardy–Weinberg equilibrium (*p* > 0.05). The minor allele frequency (MAF) of rs4778889 was significantly higher (*p* = 0.024) in the women with endometriosis (45%) compared to the reference group (31%) with odds ratio, 1.80 and 95% confidence interval (CI), 1.08 – 3.02. We also observed a statistical significance (*p* = 0.048) in the genotype frequency of rs4778889 using a recessive genetic model, further suggesting the role of rs4778889—C allele in endometriosis in Nigerian women. The distribution of genotypes and alleles of rs11556218 and rs4072111 were similar with no statistical significance (*p* > 0.05), (Table 3).

**Table 3:** Genotype and allele frequencies of Interleukin (IL)-6 and IL-16 SNPs in control and endometriosis patients

	**Control** *n* (%)	**Case** *n* (%)	***p*-value; OR (95% CI)**
**IL-6 (G > C): rs1800795**	*n* = 62	*n* = 64	
GG	62 (100)	64 (100)	-
**IL16 (C/T): rs4778889**	*n* = 62	*n* = 64	
TT	29 (46.8)	19 (29.7)	*p* = 0.08
TC	27 (43.5)	32 (50)	
CC	6 (9.7)	13 (20.3)	
TT + TC (vs. CC)^a^	56 (90.3)	51 (79.7)	*p* = 0.095, 2.38 (0.85 – 6.73)
TC + CC (vs. TT)^b^	33 (53.2)	45 (70.3)	*p* = **0.048**, 2.08 (0.98 – 4.30)
HWE (*p*-value)	0.938	0.943	
T	85 (69)	70 (55)	***p* = 0.024**
C	39 (31)	58 (45)	1.80 (1.08 – 3.02)
**IL16 (T/G): rs11556218**	*n* = 60	*n* = 62	
TT	39 (65.0)	36 (58.1)	*p* = 0.640
TG	17 (28.3)	23 (37.1)	
GG	4 (6.7)	3 (4.8)	
TT + TG (vs. GG)^a^	56 (93.3)	59 (95.2)	*p* = 0.664 0.72 (0.17 – 2.76)
TG + GG (vs. TT) ^b^	21 (35.0)	26 (41.9)	*p* = 0.431 0.75 (0.37 – 1.60)
HWE (*p*-value)	0.275	0.782	
T	95 (79)	95 (77)	*p* = 0.480
G	25 (21)	29 (23)	1.16 (0.63 – 2.13)
**IL16 (C/T): rs4072111**	*n* = 60	*n* = 62	
CC	58 (96.7)	55 (88.7)	*p* = 0.094
CT	2 (3.3)	7 (11.3)	
HWE (*p*-value)	0.896	0.638	
C	118 (98)	117 (94)	*p* = 0.121
T	2 (2)	7 (6)	3.53 (0.72 – 17.35)

*HWE* Hardy–weinberg equilibrium, *OR* Odds ratio, *CI* Confidence interval

^a^ = dominant model

^b^ = recessive model

The observed serum IL-16 concentrations were similar across the three polymorphisms investigated in IL-16 gene (rs4778889, rs11556218, and rs4072111) in both the reference group and women with endometriosis (*p* > 0.05) (Table 4).

**Table 4:** Distribution of IL-16 SNPs and serum IL-16 concentrations in both the reference and case groups

Interleukin SNP	Reference group	Case
	**Serum IL-16 (pg/ml)**	**Serum IL-16 (pg/ml)**
**IL16 (C/T): rs4778889**		
TT	23.2 [16.6 – 40.4]	20.3 [15.8 – 26.7]
TC	20.3 [16.2 – 47.6]	18.1 [16.0 – 27.9]
CC	25.2 [20.2 – 33.2]	26.8 [17.2 – 34.5]
*p*-value	0.986	0.475
**IL16 (T/G): rs11556218**		
TT	29.1 [16.7 – 46.3]	20.6 [16.0 – 28.2]
TG	24.6 [16.6 – 48.4]	17.9 [15.9 -30.0]
GG	19.6 [13.8 – 28.0]	29.9 [28.3 – 51.9]
*p*-value	0.488	0.224
**IL16 (C/T): rs4072111**		
CC	25.2 [16.6 – 46.5]	20.9 [16.2 – 31.4]
CT	15.6 [14.9 – 16.2]	15.9 [14.7 – 25.2]
TT	-	-
*p*-value	0.149	0.221

Figures presented as median [interquartile range]

**Fig. 3:**
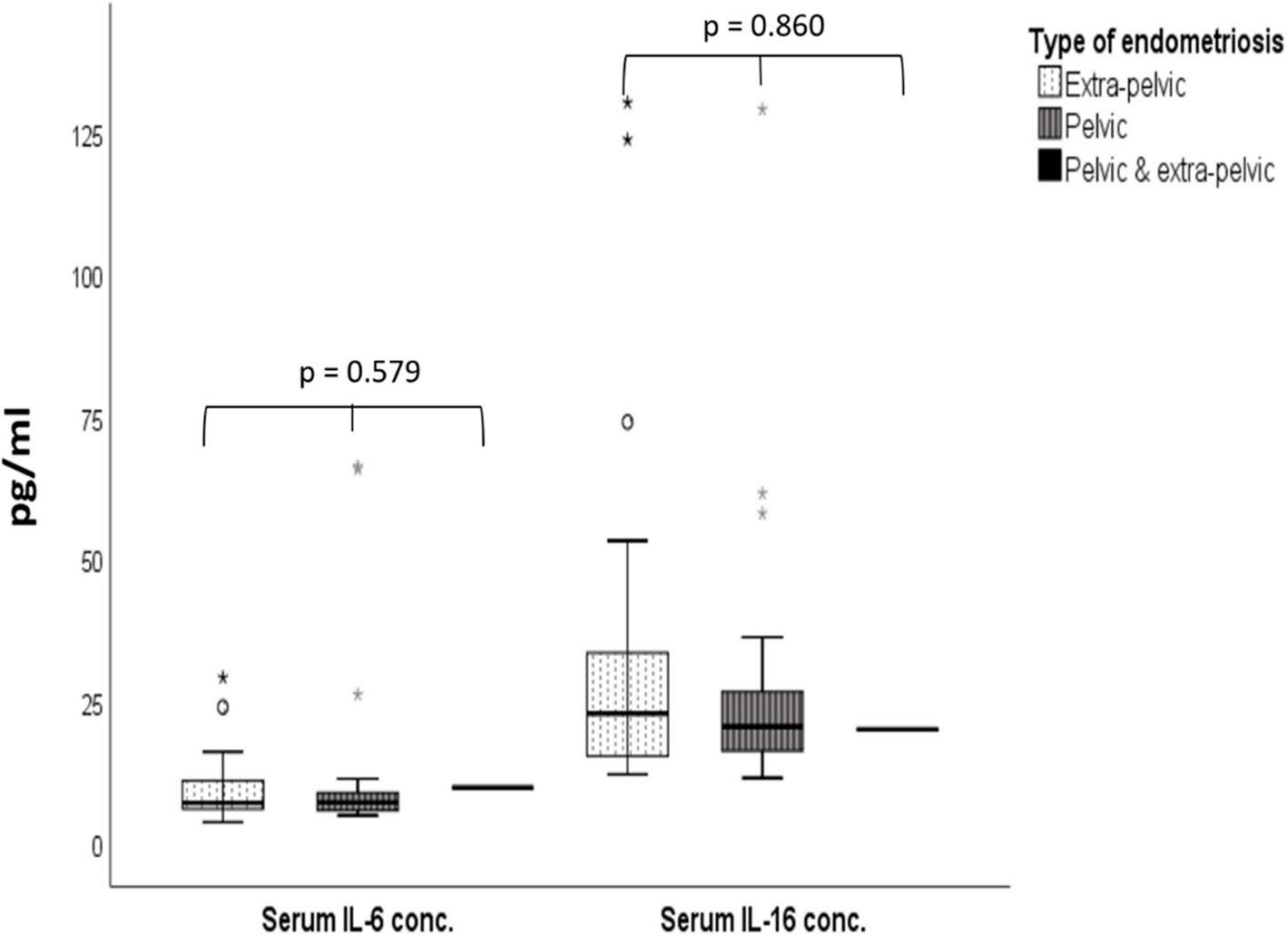
Serum concentrations of IL-6 and IL-16 according to the type of endometriosis

Serum IL-6 and IL-16 concentrations were similar in all the women with endometriosis, regardless of the clinical manifestation (Fig. 3).

Table 5 shows that pain score is not associated with polymorphisms studied in the IL-16 gene (*p* > 0.05).

**Table 5:** Pain score and IL-16 polymorphisms in women with endometriosis

	**Mild/Moderate**	**Severe**	***p*-value**
**rs4778889**			
TT	5 (23.8%)	14 (32.6%)	0.745
TC	11 (52.4%)	21 (48.8%)	
CC	5 (23.8%)	8 (18.6%)	
**rs11556218**			
TT	9 (45.0%)	27 (64.3%)	0.334
TG	10 (50.0%)	13 (31.0%)	
GG	1 (5.0%)	2 (4.8%)	
**rs4072111**			
CC	19 (95.0%)	36 (85.7%)	0.28
TT	1 (5.0%)	6 (14.3%)	

Chi square test used for hypothesis testing

## Discussion

Endometriosis affects the immune system and induce proinflammatory factors, like the cytokines. Studies on IL-6 and IL-16 in women with endometriosis have been inconsistent. Some have reported significant elevated IL-6 and IL-16 concentrations in the serum and peritoneal fluid of women with endometriosis while others did not find any statistical significance [26–29]. In this study, serum IL-6 and IL-16 concentrations were not significantly different in women with endometriosis compared to those without endometriosis. Previous studies have reported an elevated serum IL-6 concentration in women with endometriosis [28, 30, 31] but one study involving Iranian women which reported higher concentration of serum IL-6 in endometriosis failed to find a significant diagnostic value for its use in identifying women with endometriosis [27].

As expected, this study found that pain-related symptoms were commonest in women with endometriosis with most affected women experiencing dysmenorrhoea and chronic pelvic pain. This explains the exploration of association between serum IL-6 and IL-16 concentration, and IL-6 and IL-16 gene polymorphisms in women with endometriosis in this study to guide further understanding on the pathogenesis of the disease and its symptoms. Another common clinical feature was ascites, usually haemorrhagic in nature, which was reported in a third of our endometriosis cases. This presentation is not commonly reported in the literature but is seen relatively frequently in our centre, probably because we are a tertiary referral centre. A systematic review found 63% of affected women to be of African descent [32].

Women with endometriosis experiencing severe pain had significantly higher IL-16 serum concentration, but there was no association between severity of pain and IL-6 serum concentration. The lack of statistical significance might be because of the small sample size in each subgroup. There is a need to further evaluate this finding in future research on a larger population of women with endometriosis. IL-16 plays a key role in inflammation [33], and pain is a hallmark of inflammation, and this may explain the association observed between serum IL-16 and severity of pain in women with endometriosis.

The rs4778889 (T/C at position -295) in the promoter region of the IL16 gene may be associated with altered concentrations of gene expression and account for the increased concentrations of IL-16 [34]. Similar to Gan et al., [21] we observed in this study that C allele of rs4778889 was associated with endometriosis suggesting its role in IL-16 gene transcription and elevated serum IL-16 concentrations.

The rs11556218 is a missense exon-SNP (T/G), located in the exon 6 region, resulting in an amino acid change (Asparagine to Lysine) on position 446 of the Pro-IL-16, which may alter protein structure–function [9]. Contrary to our findings, Greek and Iranian women with G allele of rs11556218 of IL-16 gene had increased risk of endometriosis [7, 35].

The rs4072111 is another missense SNP (C/T: Serine to Proline) located on exon 6 of IL-16 gene [9]. We did not find association between rs4072111 and endometriosis in our study like Greek and Chinese women with endometriosis [7, 21]. This is however different from the study of Azimzade et al. where T allele showed increased risk of endometriosis in Iranian women [35].

The distribution of IL-6 rs1800795 genotypes was not associated with endometriosis but CG genotype significantly correlated with serum concentration of IL-6 in women with endometriosis [36]. rs1800795 of IL-6 was monomorphic in this current study and is consistent with the data obtained from the 1000 genome browser with G allele frequency being 1.0 in 108 and 99 persons in the Yoruba in Ibadan (YRI) and the Esan (ESN) respectively from Nigerian population [37]. Gan et al. reported a variation in the frequency of 'C' allele of interleukin 6 gene to be 4.0% in Malays, 19.0% in Indians and 0.0% in Chinese living in Malaysia [38]. Lack of polymorphism of rs1800795 seen among our subjects compared to other populations underscores how genetic diversity impacts disease susceptibility.

A large multi-centre study comprising African women is desirable to elucidate the genetic polymorphisms which underpin endometriosis.

## Conclusion

Our results revealed that elevated serum concentration of IL-16 was associated with pain severity and genetic polymorphism of IL-16 rs4778889 was associated with endometriosis in Nigerian women. Serum IL-16 and IL-16 rs4778889 may be important markers for endometriosis in Nigerian, and by extension, African women. A larger multicentre study in African women with endometriosis will provide further clarification.

## Data Availability

The datasets generated and analysed during the current study are not publicly available but are available from the corresponding author on reasonable request.
